# Population data provide evidence against the presence of a set point for hemoglobin levels or tissue oxygen delivery

**DOI:** 10.14814/phy2.14153

**Published:** 2019-06-26

**Authors:** Stephen P. Fitzgerald, Niels Grote Beverborg, Yves Beguin, Ferruh Artunc, Henrik Falhammar, Nigel G. Bean

**Affiliations:** ^1^ Departments of General Medicine and Endocrinology Royal Adelaide Hospital Adelaide South Australia Australia; ^2^ Department of Cell and Molecular Biology Karolinska Institutet Stockholm Sweden; ^3^ Department of Medicine‐Cardiology Karolinska Institutet Stockholm Sweden; ^4^ Department of Cardiology University Medical Center Groningen and Rijksuniversiteit Groningen Groningen The Netherlands; ^5^ Department of Haematology CHU de Liège and University of Liège Liège Belgium; ^6^ Department of Internal Medicine Division of Endocrinology, Diabetology, Vascular Disease, Nephrology and Clinical Chemistry University Hospital Tübingen Tübingen Germany; ^7^ Department of Endocrinology, Metabolism and Diabetes Karolinska University Hospital Stockholm Sweden; ^8^ Department of Molecular Medicine and Surgery Karolinska Institutet Stockholm Sweden; ^9^ Menzies School of Health Research and Royal Darwin Hospital Tiwi Northern Territory Australia; ^10^ School of Mathematical Sciences and ARC Centre of Excellence for Mathematical and Statistical Frontiers University of Adelaide Adelaide South Australia Australia

**Keywords:** Erythropoietin, haemoglobin, population correlations, set point

## Abstract

Hemoglobin levels are believed to be regulated as per a set point model of regulation. This model of regulation, by which specific levels of a parameter are targeted and defended by physiological systems, implies a particular population correlation between the parameter and its controlling hormone. Empirical population correlations of other parameters and their controlling hormones, have denied the presence of such set point‐based regulation. To assess if hemoglobin is regulated according to a set point model we performed a systematic search of PubMed/MEDLINE and Web of Science identifying relevant reports published up to November 2018. Population hemoglobin/erythropoietin level correlations were retrieved, and these empirically derived correlations were compared with the positive correlation implied by a set point model of regulation. Authors of papers containing potentially suitable data were contacted with requests for further analyses, and a meta‐analysis was performed. Twelve correlations between hemoglobin and erythropoietin levels from eleven papers were analyzed. None of these correlations were significantly positive, three, restricted to the normal range of hemoglobin, were significantly negative. All but one of the other correlations showed a negative trend. New analyses of previously published data sets resulted in similar findings. In particular a new analysis of large data sets of males (*n* = 2417) and females (*n* = 2592) with normal range hemoglobin levels, revealed significantly negative correlations. A meta‐analysis of our results indicated that the data overall are not consistent with a positive relationship between hemoglobin and erythropoietin (*P* < 0.0001). Population data indicate that individuals do not have set point levels of hemoglobin.

## Introduction

“Set points” are a fundamental part of models of homeostasis (Modell et al. 2015; Goldstein 2017). A “set point” is a target or ideal level of a parameter which the body defends by physiological processes, including negative feedback loops (Romanovsky 2004; Modell et al. 2015). Set points have been likened to thermostat or cruise control settings (Modell et al. 2015). A variety of parameters, including body temperature, blood levels of sodium, glucose, and calcium have been described as being so regulated, that is, as per a “set point model”(Modell et al. 2015).

Hemoglobin and tissue oxygen delivery are also considered to be regulated as per a set point model (Patel 2008; Patel et al. 2009; Murphy et al. 2010; Bachman et al. 2014). Certainly, there is little intra‐individual (within ‐ person) variation in hemoglobin levels (Brown and Goodall 1946). Men and women (Murphy et al. 2010), and individuals of the same sex across different races/ethnicities (Patel 2008) are considered to have different set points for hemoglobin.

Alternative models of homeostasis include the “balance point model” whereby the level of the parameter is not pre‐set but settles at the point of balance of the regulatory physiological processes (Romanovsky 2004). Conventional teaching is that cardiac stroke volume is such a parameter (Klabunde 2005). The “settling point model” (Speakman et al. 2011) is another model of regulation, this model again lacking a set point.

The absence of a set point does not imply that parameters homeostatically regulated do not settle around a level which tends to be stable (Romanovsky 2004).The determination as to whether a set point exists or not cannot be readily determined by physiological experimentation. The stability of the parameter level and the responses of the associated physiological regulating factors may be identical with or without the inclusion of a set point in the model of regulation (Romanovsky 2004).

Studies of population data may be more helpful in this area. For example it has been argued that the increasing population prevalence of obesity, the tendency for obesity to occur in the least affluent members of society, and the associations of obesity with television watching, marriage and college attendance all argue against the presence of a set point that determines weight (Speakman et al. 2011).

Similarly, we have previously shown that a set point model of regulation implies a particular population (between ‐person) correlation between a parameter and its regulating hormone, that is, the population correlation should be in the direction of the effect of the controlling hormone on the parameter (Fitzgerald et al. 2017; Fitzgerald and Bean 2018a). (The concept of an adjustable set point (Cabanac 2006) does not affect this property of a set point model.) For example, if regulation were according to a set point model, for glucose the population correlation should be negative (insulin lowers the blood glucose level), and for calcium it should be positive (parathyroid hormone raises the serum calcium level) (Fitzgerald and Bean 2018a).

The reason why the correlations should be as described in a set point model is presented fully in our previous work (Fitzgerald et al. 2017; Fitzgerald and Bean 2018a). Heuristically, the principle can be understood as follows, using the subject of this work, the hemoglobin/erythropoietin system as the example.

Physiologically, hemoglobin levels are controlled by erythropoietin via a negative feedback loop (Jelkmann 2011; Bunn 2013). Strictly speaking erythropoietin controls oxygen delivery to tissues (Jelkmann 2011; Bunn 2013), this oxygen delivery being a product of the oxygen tension and hemoglobin level in the blood. However, in a normal population of individuals, all living at the same altitude, there is a uniform oxygen tension in terms of erythropoietin physiology, and so in these circumstances erythropoietin is responsible for maintaining hemoglobin levels (Jelkmann 2011; Heuberger et al. 2013).

According to the set point model, in a physiologically normal population there will be a range of normal hemoglobin values, corresponding to a range of normal set points. There thus will be individuals with either relatively high or relatively low set points of hemoglobin levels. Given that the hemoglobin levels are controlled by the levels of erythropoietin, the only way the physiologically normal individuals with the relatively high set points of hemoglobin can attain the high levels of hemoglobin is by having relatively high levels of erythropoietin, whereas the individuals with the relatively low set points for hemoglobin must keep their erythropoietin levels relatively low so as to avoid a hemoglobin level above their set point level. There is thus generated a positive population, between ‐person, relationship between hemoglobin and erythropoietin levels. Note that in each individual in this population there is still a negative relationship between hemoglobin and erythropoietin in terms of individual, within‐person, responses to alterations to the hemoglobin level.

Given therefore that the set point model implies a certain population correlation between a parameter and its controlling hormone it is possible to test the validity of the model for any parameter by comparing the implied correlation with that derived by empirical observation. Our previous work, using this method of investigation, indicated that, contrary to conventional teaching, blood levels of calcium, glucose (Fitzgerald and Bean 2018a) and thyroxine (Fitzgerald et al. 2017) are not regulated as per a set point model.

The aim of this study was to determine whether or not empirical population correlations between hemoglobin and erythropoietin are consistent with the positive correlation implied by the set point model.

## Methods

We systematically reviewed the published literature for empirical data concerning the population correlation between hemoglobin and erythropoietin. Furthermore, when we found papers describing data and analyses that did not pertain solely to the normal range we contacted the authors of these papers and requested further data and/or analyses.

### Search strategy

A systematic search of PubMed/MEDLINE and Web of Science using terms such as erythropoietin, hemoglobin/hemoglobin, population, and correlation was conducted to identify relevant articles published up to November 2018. No restrictions were placed on language, country, or publication date. Initially the titles of the articles were screened for relevance and then the abstracts, with full‐text reports of potentially relevant reports reviewed. Additional relevant articles were searched for in the reference lists of the retrieved full‐text studies. The literature search was conducted independently by two of the authors (SPF and HF), and the included and excluded articles were agreed on by consensus with reference to the criteria described in the next section.

### Study selection and data extraction

Studies reporting on population hemoglobin and erythropoietin level correlations were included. Reports were excluded if data was not to be considered a reflection of, at least in part, a normal population (i.e., free of anaemia, polycythemia and disease affecting erythropoietin physiology), or the studied population was less than 20 individuals. Review articles, editorials, and meeting abstracts were also excluded.

We extracted from each study the empirically derived associations between hemoglobin and erythropoietin levels, the form of the expression of this association (e.g., Pearson correlation, slope of line of best fit), and the statistical significance of any association. The following information was also extracted from each study: first author, number of individuals in the population, sex, and the prevalence of anemia. We did not address the individual methodology of hemoglobin and erythropoietin measurement on the grounds that any correlations would remain similar with any valid laboratory method.

The use of a quality assessment (e.g., the Newcastle‐Ottawa Scale; available at: www.ohri.ca/programs/clinical_epidemiology/oxford.asp) was considered inappropriate because these instruments had not been developed for this setting. The PRISMA (Preferred Reporting Items for Systematic Reviews and Meta‐Analyses) guidelines were followed (Moher et al. 2009).

### Data analysis

The empirically determined correlations were compared with the positive correlation implied by the set point model. Only the empiric correlations within the normal range for the parameter were considered. The evidence for the correlations within the normal range was considered strongest when the correlations were separately or only calculated for data lying within the normal range. When the line of correlation passed through the normal range but this line had possibly been affected by consideration also of data lying outside of the normal range we considered the evidence for the correlations within the normal range to be less strong.

We performed a meta‐analysis of Pearson correlation coefficients (using *metacor* from the R‐package *meta* (R Core Team, 2017; Schwarzer 2007) with Fisher's *z* transformation of correlations and a fixed‐effects model as indicated by the in‐built test of heterogeneity) and a forest plot to test whether the collection of published results were consistent with a positive relationship between hemoglobin and erythropoietin. In order to include the small studies that did not provide Pearson correlation coefficients we also conducted a more general meta‐analysis that did not require Pearson correlation coefficients per se, merely the sign of the slope of the linear relationship and its statistical significance. To do this we classified all outcomes in one of four categories:

- positive relationship that is statistically significant,
- positive relationship that is not statistically significant,
- negative relationship that is not statistically significant, and
- negative relationship that is statistically significant.

We used the conservative null hypothesis of no population‐level relationship between hemoglobin and erythropoietin (we cannot handle an unspecified positive relationship) and regarded the different papers as independent observations of this relationship. As all the papers had used a significance level of 5%, the expected proportion of results to fall in these four categories should be 2.5%, 47.5%, 47.5%, and 2.5%, respectively. We therefore conducted exact multinomial tests (using *multinomial.test* from the R package *EMT* (Menzel 2013)) on all of our results, regardless of the manner of their reporting, to test whether they were consistent with the above proportions and hence the null hypothesis of no relationship.

As our data included analyses which were not restricted to the normal range of hemoglobin, we also conducted the same process on the restricted set of analyses that had been classified as only considering the normal range. When we in addition obtained new analyses, conducted on the original data with additional restrictions as to the normality of subjects, we tested these analyses and also tested the combination of the results from the systematic review, restricted to the normal range of hemoglobin, and the results of the new analyses.

## Results

**Figure 1:**
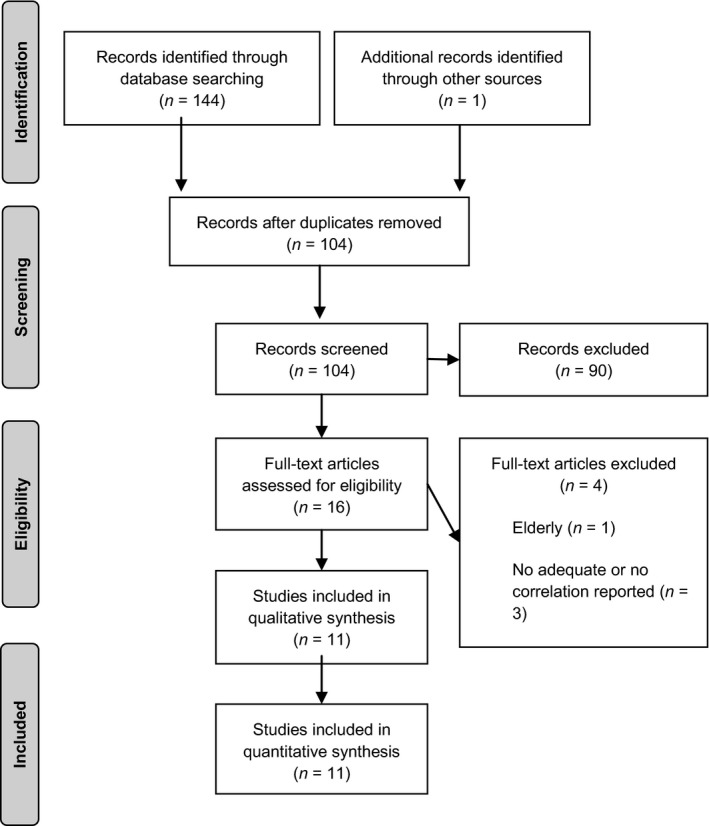
Flowchart illustrating the procedure for article inclusion and exclusion in a systematic review of set point for hemoglobin level. A systematic search of PubMed/MEDLINE and Web of Science up to November 2018, with manual searching of reference lists, identified only 11 relevant reports.

The systematic search identified 144 potentially relevant records. Another study was identified through review of the reference lists. After removal of duplicates, 104 records were screened for eligibility, identifying eleven data sets suitable for analysis (Beguin et al. 1991, 1993; Carretti et al. 1993; Schrieber et al. 1996; Charuruks et al. 2000; Roque et al. 2001; Fehr et al. 2004; Ershler et al. 2005; Artunc and Risler 2007; Mercadal et al. 2012; Grote Beverborg et al. 2015) (Fig. 1). Three studies were most suitable as they specifically examined the normal range (Beguin et al. 1991; Charuruks et al. 2000; Mercadal et al. 2012). We found one further report (Ershler et al. 2005) dealing with the normal range but measures of significance were not supplied. We included a small study concerning erythropoietin in pregnant individuals (Carretti et al. 1993) accepting that these subjects may, or may not be considered normal. We also found six studies that included a correlation for hemoglobin and erythropoietin through the anemic and normal ranges (Beguin et al. 1993; Schrieber et al. 1996; Roque et al. 2001; Fehr et al. 2004; Artunc and Risler 2007; Grote Beverborg et al. 2015).We did not include two further studies (Malagarnera et al. 1996; Panjeta et al. 2017) reporting correlations between renal function and erythropoietin, and renal function and hemoglobin but not directly between hemoglobin and erythropoietin.

All of the above data sets had originally been developed for reasons unrelated to the topic of this paper.

The results of our literature search are summarized in Table 1.

**Table 1:** Summary of published studies indicating a correlation between hemoglobin (Hb) and Erythropoietin (Epo)

Author/year	Population	Sex M = male, F = female/n/abnormal Hb	Normal Hb range separately considered Y=Yes, N = No	Correlation *r* = Pearson correlation coefficient *S* = slope log Epo vs Hb	Significance
Charuruks et al. (2000)	Blood donors	F/100/0	Y	*r* = +0.015	NS
		M/100/0	Y	*r* = −0.289	*P* < 0.01
Mercadal et al. (2012)	GFR^c^ >30	M and F/204/0	Y	*r* = −0.05	*P* = 0.58
Ershler et al. (2005)	Normal population	M 54%/143/0	Y	‘Negative’ Not specified further	‘significant’ not specified further
Beguin et al. (1993)	Normal + blood disorders not affecting Epo	Not specified/195/38 polycythemia,126 anemia	Y (Hct > 40%)	*S* = −0.003	NS
Carretti et al. (1993)	pregnant	F/96/0	Y (Hb > 10.5 g/dL)	*r* = −0.114 a	NS
Grote Beverborg et al. (2015)	General Population GFR > 60	M 50.1%/6777/9.4% anemia	N	power = −2	*P* < 0.001
Roque et al. (2001)	Healthy volunteers	M and F/100/50	N	*S* = −0.04275	*P* < 0.0001
Fehr et al. (2004)	CCl^b^ >40	M 60%/289/119 anemia (Hb < 13G/dL)	N	*r* = −0.35	*P* < 0.001
Artunc and Risler (2007)	Anemia work‐up/No CKD^c^	M 49%/167/21% anemia	N	*S* = −0.135	*P* < 0.0001
Schrieber et al. (1996)	No IBD^c^ (controls)	Not specified/42/18 anaemia	N	*S* = −0.0522	*P* < 0.001
Beguin et al. (1991)	Normal + anemia	Not Spec./166/82 anemia	N	*S* = −0.0695	*P* = 0.000

Glomerular filtration rate.

a correlation with haematocrit (Hct) rather than Hb.

Creatinine clearance.

b Chronic Kidney disease.

c Inflammatory bowel disease.

None of the five studies (including the study of pregnant women) specifically examining the normal range, or the six studies examining the normal range in conjunction with the anemic range, revealed a significantly positive correlation between hemoglobin and erythropoietin. Significantly negative correlations were found. None of the studies therefore support the existence of a set point for hemoglobin and each of the data sets, to a greater or lesser extent, are in fact inconsistent with the existence of such a set point. By contrast, all of the data sets are consistent with a balance point model of hemoglobin regulation.

We were able to have further analyses performed on three of the above‐mentioned studies by the original authors (Table 2). The results were qualitatively similar. When considering the normal range of hemoglobin, the two smaller studies (Beguin et al. 1991; Artunc and Risler 2007) between them showed non‐significant negative correlations. One of these re‐analyses (Beguin et al. 1991) reconsidered the analysis performed earlier, rather than examining a significantly different sub‐group. To avoid double counting, only this second analysis was considered when we performed a meta‐analysis including studies from both Tables 1 and 2.

**Table 2:** Summary of new analyses of hemoglobin (Hb) and Erythropoietin (Epo) on previously obtained data

Author/year	Population	Sex/n	Normal Hb range separately considered Y = Yes, N = No	Correlation *r* = Pearson correlation coefficient	Significance
Grote Beverborg et al. (2015)	General population	Male/2417	Y (14–17.5 g/dL)	*r* = −0.084	*P* < 0.0001
		Female/2592	Y (12.3–15.3 g/dL)	*r* = −0.1174	*P* < 0.0001
Artunc and Risler (2007)	Non anaemic subset of total sample	Not specified/29	Y (12–16 g/dL)	*r* = −0.2868	*P* = 0.5167
Beguin et al. (1993)	Reference set	Not specified/21	Y (Hct 38.5–51)	*r* = −0.0493^a^	*P* = 0.83

Correlation with Hct rather than Hb.

**Figure 2:**
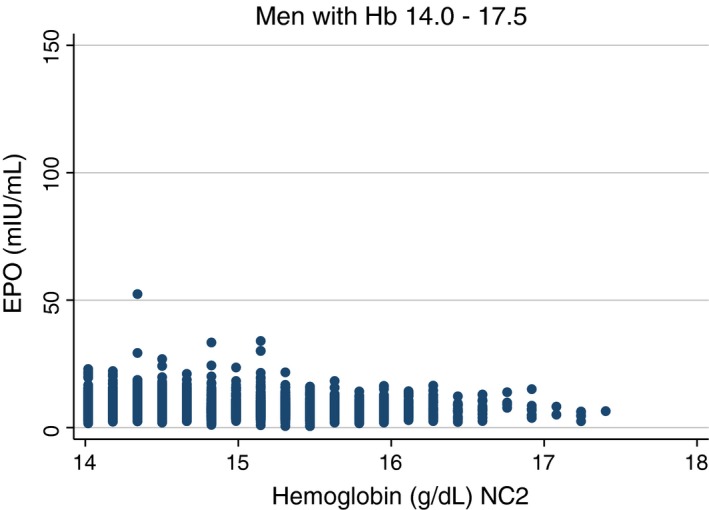
Scatter plot of simultaneous hemoglobin and erythropoietin levels, restricted to hemoglobin values in the normal range, in men of the general population study of Grote Beverborg et al. (2015).

**Figure 3:**
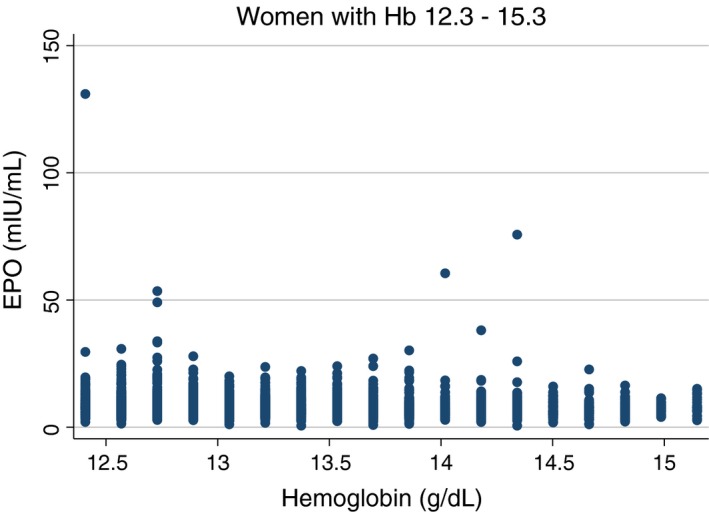
Scatter plot of simultaneous hemoglobin and erythropoietin levels, restricted to hemoglobin values in the normal range, in women of the general population study of Grote Beverborg et al. (2015).

More importantly re‐examination of Grote Beverborg's large general population study (Grote Beverborg et al. 2015) revealed highly significant (*P* < 0.001) negative correlations between hemoglobin and erythropoietin in individuals of both sexes with normal range haemoglobin levels (Table 2, Figs. 2 and 3). Furthermore, the highly significant negative correlation persisted when the populations were restricted by excluding the presence of any of a number of complicating medical conditions (Table 3). Finally, as the lower limit of hemoglobin was raised to exclude the possibility of ever more subtle disease the negative correlations persisted albeit with attenuated significance (Table 3).

**Table 3:** Sensitivity studies, with increasingly strict hemoglobin (Hb) range, of correlation between hemoglobin (Hb) and Erythropoietin (Epo) in reference set population of Grote Beverborg et al. (2015)

Sex/n	Hb range	*r* = Pearson correlation coefficient	Significance
Male/1092	Hb > 13 g/dL	*r* = −0.1046	*P* = 0.0005
Female/1149	Hb > 12 g/dL	*r* = −0.1436	*P* < 0.0001
Male/816	14–17.5 g/dL	*r* = −0.0670	*P* = 0.0556
Female/997	12.3–15.3 g/dL	*r* = −0.1226	*P* = 0.0001
Male 817	>14 g/dL	*r* = −0.0062	*P* = 0.0586
Female 942	>12.5 g/dL	*r* = −0.1163	*P* = 0.0003
Male 561	>14.5 g/dL	*r* = −0.0483	*P* = 0.2537
Female 623	>13.0 g/dL	*r* = −0.0715	*P* = 0.0723

a Reference set‐ exclusion criteria‐ CRP > 5, GFR < 60, presence of anemia (Hb < 12 female, Hb < 13 male), presence of heart failure, BMI > 30, COPD, asthma, UAE > 10 mg/L at follow‐up.

**Figure 4:**
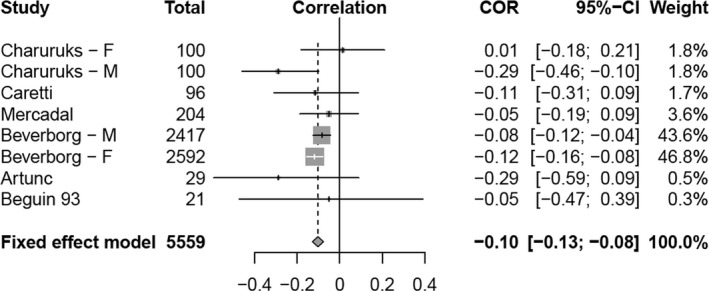
Forest plot of all studies with Pearson correlation coefficients, of subjects with normal Hb

When we subjected our results to correlation coefficient meta‐analysis we were only able to combine the studies that provided Pearson correlation coefficients. Including only the studies on individuals with normal hemoglobin from Table 1 resulted in a non‐significant negative correlation. When the data were enriched by the new analyses in Table 2 we found a confidence interval for the correlation to be −0.1269 to −0.0747, *P* < 0.0001. A forest plot (Fig. 4) confirmed the negative correlation overall.

The small studies that did not provide Pearson correlation coefficients provided results in the same direction as the overall value and their inclusion would not be expected to materially change the results. In particular the only study so missing from analysis of studies pertaining solely to the normal hemoglobin range was that of Ershler et al. (2005) which reported a significantly negative correlation.

The exact multinomial tests conducted on all of our results, regardless of the manner of their reporting, confirmed our coefficient meta‐analyses and forest plot. We first considered all of the results from Table 1 and found *P* < 0.0001. Consideration of only the results from Table 1 pertaining to the normal range resulted in *P* = 0.013. The analysis of results from Table 2 resulted in *P* = 0.0039 and examination of all the results from Tables 1 and 2 pertaining to the normal range only, resulted in *P* < 0.0001. Thus, we consistently reject the null hypothesis of no population‐level relationship and are therefore left with the alternative hypothesis that there is in fact a negative population‐level relationship between hemoglobin and erythropoietin.

## Discussion

We have found that the literature generally states that there is a set point for hemoglobin (Patel, 2008; Patel et al. 2009; Murphy et al. 2010; Bachman et al. 2014 ) and that the physiological correlation between hemoglobin and erythropoietin is negative (Beguin et al. 1991; Carretti et al. 1993). It has not been appreciated that these two premises are mutually exclusive. The understanding of the negative correlation has been based on small studies and on studies combining normal subjects with those with abnormal hemoglobin levels.

The new analyses we have presented provide stronger evidence of the negative population correlation between hemoglobin and erythropoietin within the normal range. Our work thereby also provides evidence against the presence of a set point for hemoglobin. Our conclusion applies equally to the parameter of tissue oxygen delivery. Therefore these parameters may be added to a list that includes body temperature, and blood levels of thyroxine, glucose, and calcium, of parameters unlikely to be regulated as per a set point model.

The methodology and logic of this current work is similar to that of our previously published work (Fitzgerald et al. 2017; Fitzgerald and Bean 2018a). This work differs in that addition to reviewing the literature we were able to generate new correlations by re‐analysis of previously presented data. The eleven data sets upon which we base our conclusions regarding hemoglobin and erythropoietin provide strong evidence. In a set point model, as allowed by statistical variation, all data sets should be consistent with the model; thus, even if additional data sets were discovered to have a positive relationship and hence be consistent with a set point model, the data sets we found would still stand as evidence against the existence of a hemoglobin set point. Our general meta‐analysis, using the exact multinomial tests, is very conservative as the null hypothesis was the absence of a correlation rather than a positive correlation.

The studies that considered the correlation between hemoglobin and erythropoietin across the anemic and normal ranges *concurrently* potentially might be showing a negative correlation across the normal range on account of the points in the anaemic range. Nevertheless even within these studies there is some evidence of a negative relationship, and no strong evidence of a positive relationship, within the normal range. In particular the correlation between hemoglobin and erythropoietin remained negative in populations with high numbers of individuals with normal hemoglobin levels and minimal numbers of significant anaemia. It seems implausible that the low numbers of anaemic individuals could change a population correlation not just from a positive correlation to no correlation, but further to a negative correlation.

The studies that specifically examined the normal range, and most particularly the re‐analysis of Grote Beverborg's data sets (Grote Beverborg et al. 2015), provide the strongest evidence of a negative correlation between hemoglobin and erythropoietin. Grote Beverborg's re‐analysis approaches an ideal study, and might well stand on its own. It is large, the analysis is restricted to normal individuals as strictly defined, and in particular excludes individuals with conditions likely to interfere with the assessment of the normal relationship between hemoglobin and erythropoietin. It also includes supporting sensitivity analyses. The previously available analyses restricted to normal individuals, were hampered by, a less strict definition of normal, the limited analyses, and/or the small sizes of the population studied.

The totality of evidence therefore points to a negative relationship between hemoglobin and erythropoietin which strengthens from the normal range into the anemic range.

Our conclusions rely on the validity of the empiric data. It would, however, require great corruption of the normal range data, for example, via a proposal that the normal range is corrupted by great numbers of pathological individuals not at set point, to reverse the correlations. Any corruption based on significant cardiorespiratory conditions affecting tissue oxygen delivery would tend to strengthen a positive population correlation between hemoglobin and erythropoietin rather than reverse it. Any corruption in terms of occult anemia would need to be in all data sets, including Grote Beverborg's carefully selected reference sets (Grote Beverborg et al. 2015), and resist our progressive lifting of the lower limit of the examined hemoglobin range.

There is however no reason to posit such a proposition of data corruption, which would also negate all the previous work suggesting that hemoglobin set points differ across sex and race, just as there is no reason to propose unidentified physiological mechanisms that also might alter population correlations.

It is interesting that of all of the data sets we have now found, for thyroxine (Fitzgerald et al. 2017), glucose and calcium (Fitzgerald and Bean 2018a), as well as for hemoglobin, none have revealed correlations supportive of a set point model. The correlations of the larger studies have not contradicted the set point model by being merely neutral; they have tended to be significantly in the opposite direction to that implied by the model. The fact that the evidence for all these parameters is inconsistent with set point based physiology also supports the evidence for each; that is, the evidence we have presented regarding the regulation of hemoglobin does not represent a physiological or statistical aberration. To gainsay the totality of evidence one must posit that not only the normal range for hemoglobin is corrupted or that there are other unidentified physiological processes at play in this system, but that the same applies for the other parameters.

The pattern of population correlations suggests in fact that there is a general pattern in physiological regulation, by which the population variation in the controlling hormone physiology (e.g., erythropoietin response to hemoglobin), is less than the population variation in the parameter physiology (e.g., hemoglobin response to erythropoietin) (Fitzgerald and Bean 2016). In the circumstance of a given variation in parameter physiology, such an arrangement leads to a minimisation in intra‐, and inter‐individual variation in parameter levels (Fitzgerald and Bean 2018b). Such an outcome is particularly advantageous with the parameter, hemoglobin.

In conclusion, the evidence indicates that individuals do not have set point levels of hemoglobin. Apart from the clarification of the physiology of individual parameters, the progressive discovery of parameters not regulated as per a set point model, casts increasing doubt on the concept that regulation by a set point model is the norm in physiology.

This clarification does not affect the validity of other work detailing the physiological mechanisms involved in homeostasis or important in adaptive physiology (for example adaptation to altitude). In particular our work does not contradict the evidence that erythropoietin levels rise in the event of a drop in hemoglobin levels such that a stable level of hemoglobin is restored. Our work does aid physiological research as it explains the difficulties finding the physiological substrates of set points (Modell et al. 2015). It enables clearer explanations as to why hemoglobin levels tend to differ between sexes and races. It also re‐frames the discussion of normal ranges of various parameters, for example, there is no need to propose that an individual has an individually set level of a parameter such that the individual is disadvantaged by a shift from this level even though this shifted level may still lie within the population normal range (Andersen et al. 2002).

Further practical consequences of this advance in the comprehension of the fundamental outline of physiological regulation may emerge.

## Conflict of Interest

There were no conflicts of interest for any of the authors in regard with this work.
